# The Other Side of the Vaccination Question

**Published:** 1902-02-15

**Authors:** 


### THE OTHER SIDE OF THE VACCINATION QUESTION.

The Chicago health officials do not hesitate to proclaim the faith that is in them. They have issued "a vaccination creed" declaring their personal belief in vaccination, and this being printed and hung up for public inspection in lodging houses, factories, workshops, etc., is said to have done much to give confidence to the people. This creed, however, also contains certain paragraphs which are calculated to make some people very seriously question whether their vaccination has been properly done. For example, it defines "true vaccination" as vaccination which has been "repeated until it no longer takes," which is certainly not the sort of vaccination to which many people in this country have submitted themselves. Then it says that true vaccination "never did and never will make a serious sore," which again must lead many to believe, and probably correctly, that their vaccination has been anything but "true," according to this definition. By the insertion of a parenthesis, however, we are told that true vaccination is "vaccination properly done, on a clean arm, with pure lymph, and kept perfectly clean and unbroken afterwards;" the latter provision reminding one rather of the old plan of catching sparrows by putting salt on their tails. We have no doubt at all that when the vesicle does not break there will be no serious sore; the difficulty is when the vesicle does break. We have every sympathy with those who wish to extend the practice of vaccination, but these public statements to the effect that when properly done vaccination never produces a serious sore, and that a man who has been truly vaccinated will "take" no longer, are likely to land medical men in many difficulties. The public will not be slow to perceive that such statements involve a good deal. For example, they would appear to involve the corollary that if a serious sore is produced it is the fault of someone, presumably the doctor; and that, if after being successfully vaccinated someone else is able to produce a fresh crop of vesicles, the first vaccination has not been properly performed. If such statements are once accepted as defining true vaccination, the medical defence unions will soon find themselves fully occupied in defending their clients from actions for malpraxis.

